# Validity of Heart Rate Measurement Using Wearable Devices During Cardiopulmonary Exercise Testing in Patients With Cardiovascular Disease: Prospective Pilot Validation Study

**DOI:** 10.2196/77911

**Published:** 2025-10-06

**Authors:** Kazufumi Kitagaki, Yuji Hongo, Rie Futai, Takeshi Hasegawa, Hiroshi Morikawa, Hisashi Shimoyama

**Affiliations:** 1 Faculty of Rehabilitation, Shijonawate Gakuen University, 5-11-10 Houjou Daito, Osaka, 574-0011, Japan, 81 72-863-5043, 81 72-863-5022; 2 Department of Medical Technology, Itami City Hospital, Itami, Japan; 3 Department of Cardiovascular Medicine, Itami City Hospital, Itami, Japan

**Keywords:** validation, heart rate measurement, wearable device, Fitbit Inspire 3, heart failure

## Abstract

**Background:**

Wearable devices offer a promising solution for remotely monitoring heart rate (HR) during home-based cardiac rehabilitation. However, evidence regarding their accuracy across varying exercise intensities and patient profiles remains limited, particularly in populations with cardiovascular disease (CVD) such as those with heart failure (HF).

**Objective:**

The objective of this study was to evaluate the accuracy of HR measurements obtained using the Fitbit Inspire 3 during cardiopulmonary exercise testing (CPX) in patients with CVD, including those with HF.

**Methods:**

In this single-center, prospective pilot study, we enrolled 30 patients with CVD undergoing CPX. HR was simultaneously recorded using electrocardiography and the Fitbit Inspire 3 at 1-minute intervals across various CPX phases: rest, exercise below and above the anaerobic threshold (AT), and recovery. The correlation between the two methods was assessed using the Pearson correlation coefficient. Measurement error was quantified by mean absolute error and mean absolute percentage error (MAPE), with a MAPE of ≤10% defined as the threshold for acceptable agreement.

**Results:**

All data points were 630 points per minute. The Fitbit Inspire 3 device demonstrated a strong overall correlation with electrocardiography-derived HR (*r*=0.90; IQR 0.88‐0.91) and an acceptable MAPE of 5.40% (SD 8.33%). The total error was 14.9% (94/630), with overestimation and underestimation of 37 (5.8%) points and 57 (9%) points, respectively. The rate of HR underestimation reached 19 (16%) points during exercise above the AT, compared to 1 (3%) point at rest. When stratified by HF stage (B vs C), underestimation was more pronounced in patients with HF (14/275, 5% points vs 40/355, 11.2% points).

**Conclusions:**

The Fitbit Inspire 3 provides acceptable validity for HR monitoring during CPX in patients with CVD. However, clinicians should interpret HR data with caution during high-intensity exercise, especially in patients with HF.

## Introduction

### Background

Outpatient cardiac rehabilitation (CR) is a class I recommended therapy for patients with cardiovascular disease (CVD) and is regarded as an essential component of treatment [1]. However, in Japan, the prevalence of heart failure (HF) is rising among older adults [2], while participation in outpatient CR remains low [3]. Among working-age patients with coronary artery disease (CAD), the challenge of balancing work and CR contributes to the low participation rates [4]. A recent meta-analysis [5] demonstrated that home-based CR combined with digital support yielded comparable improvements in quality of life and reductions in hospital readmissions to conventional outpatient CR. These findings highlight the potential significance of remote support in home-based CR programs.

Maintaining appropriate exercise intensity is crucial to prevent symptom exacerbation when prescribing exercise therapy [1]. Heart rate (HR), closely linked to oxygen consumption [6], is a key indicator for setting exercise intensity and is commonly used in outpatient CR [7]. HR monitoring during outpatient CR is typically performed using electrocardiography (ECG). However, in home-based settings, continuous ECG monitoring is impractical, leading to reliance on manual pulse checks, whose accuracy remains insufficiently validated.

Advances in wearable digital technology have led to the widespread use of devices using photoplethysmography (PPG), such as smartwatches, which are being investigated as potential alternatives to ECG [8]. A recent scoping review suggested that smartwatches may be useful tools to support and enhance outpatient disease management [9]. However, measurement accuracy can vary widely between devices [10], and HR obtained via wearable devices tends to be underestimated at higher exercise intensities [11]. Consequently, whether such technology can be reliably applied to patients with CVD, particularly those with HF who may experience reduced peripheral perfusion, warrants careful evaluation.

A recent validation study [12] reported that HR measurements using the Apple Watch 7 (Apple Inc) and Galaxy Watch 4 (Samsung Electronics Co, Ltd) during cardiopulmonary exercise testing (CPX) were highly accurate in patients with CAD, indicating their potential utility in this population. However, these devices are relatively expensive (approximately US $350), and their accessibility to individuals with low income, who may already be reluctant to participate in outpatient CR, remains limited [4]. Moreover, although the study population included stable patients with CAD, the accuracy of measurement in patients with HF was not evaluated.

### Objective

We hypothesized that if a more affordable device, such as the Fitbit Inspire 3 (Fitbit LLC; approximately US $80), could yield similar HR measurement accuracy, it would become more widely accessible. Previous research on the Fitbit series in postoperative patients performing low-intensity activities [13] showed promising accuracy, and a validation study involving younger patients with CVD demonstrated good performance during stepwise increases in exercise intensity measured by CPX [14]. However, no studies have specifically focused on patients with HF. Therefore, this study aimed to evaluate the accuracy of HR measurement from the Fitbit Inspire 3 compared to ECG-based measurements during CPX in patients with CVD, including those with HF.

## Methods

### Study Design and Setting

This single-center, prospective, observational pilot study was conducted at the Itami City Hospital.

### Participants

Participants were recruited from patients who received a prescription for CPX at the Department of Cardiology, Itami City Hospital, between August 2024 and March 2025. The inclusion criteria were as follows: (1) aged ≥18 years at the time of consent, (2) documented agreement to undergo CPX, and (3) provision of written informed consent for study participation. The exclusion criteria were as follows: (1) aged <18 years, (2) persistent atrial fibrillation (AF), (3) known silicone allergy, or (4) refusal to participate.

### Sample Size Calculation

As a pilot study, a target sample size of 30 participants was established based on previous studies with sample sizes ranging from 10 to 60 [10], as well as the estimated number of CPX procedures expected during the study period.

### Data Collection and Procedures

CPX was performed using a cycle ergometer equipped with a breath-by-breath gas analyzer (AE-300S; Minato Medical Science). The testing protocol included a 1-minute rest period and 4 minutes of warm-up at 0 W, followed by a symptom-limited ramp protocol with individualized increments of 10‐20 W/min. The recovery phase involved pedaling at 0 W for ≥5 minutes until clinical status stabilized. Ventilatory parameters, including minute ventilation (VE), oxygen uptake (VO₂), and carbon dioxide output (VCO₂), were recorded every 6 seconds.

The respiratory exchange ratio was calculated as VCO₂/VO₂ using breath-by-breath data. Peak VO₂ was defined as the highest VO₂ value recorded during exercise or the average of the final 18 seconds (3 data points), whichever was greater. Peak VO₂ was normalized by body weight (BW) and expressed in mL/kg/min (peak VO₂/BW), in accordance with standard clinical practice for CPX. The anaerobic threshold (AT) was determined by an experienced cardiologist based on multiple indices: the inflection point in VE/VO₂ without a concurrent rise in VE/VCO₂, increasing partial pressure of end-tidal oxygen without a change in end-tidal carbon dioxide, and other standard criteria.

During CPX, the Fitbit Inspire 3 device was attached to the arm contralateral to the side used for blood pressure measurement. The device was worn approximately 2 finger-widths above the wrist crease, with the optical sensor positioned flush against the skin to minimize motion artifacts. HR was recorded simultaneously using ECG and the wearable device, which logged data at 1-minute intervals [15]. Test start times were extracted from the electronic medical records. HR data from the wearable device were retrieved through the Fitbit Web Application Programming Interface using the *httr* and *jsonlite* packages in R (version 4.4.0; R Foundation for Statistical Computing).

### Demographic and Clinical Characteristics

All variables were obtained from patients’ medical records. Baseline demographics data included age, sex, diagnosis, American College of Cardiology (ACC) or American Heart Association (AHA) HF stages [16], relevant medical history, current medications, and smoking status. Physical examination findings included height, weight, BMI, and the New York Heart Association functional classification. Laboratory parameters included hemoglobin, creatinine, estimated glomerular filtration rate, and B-type natriuretic peptide. Echocardiographic data included left ventricular ejection fraction (LVEF), measured using the modified Simpson method. HF phenotypes were categorized as HF with reduced ejection fraction (LVEF≤40%), HF with mildly reduced ejection fraction (LVEF 41%‐49%), and HF with preserved ejection fraction (LVEF≥50%) [17].

### Statistical Analysis

Continuous variables are presented as mean (SD) or median (IQR), as appropriate. Categorical variables are expressed as counts and percentages.

A whole-test analysis was performed using the complete set of 1-minute HR data. In addition, a phase-specific analysis was conducted by assigning each 1-minute sample to 1 of 4 predefined exercise phases: rest, low-intensity (including warm-up; below AT), high-intensity (above AT), and recovery. A stratified whole-test analysis was also carried out based on the ACC or AHA HF stage classification.

Furthermore, stratified analyses by ACC or AHA stage were conducted by repeating both the whole-test and phase-specific analyses within each stage category. Pearson correlation coefficients were calculated to assess the relationship between ECG-based and Fitbit-derived HR. Correlation coefficients were interpreted as follows: 0‐0.30 as negligible, 0.30‐0.50 as low, 0.50‐0.70 as moderate, 0.70‐0.90 as high, and 0.90‐1.00 as very high. Bland-Altman plots were used to evaluate agreement, systematic bias, and limits of agreement. Mean absolute error (MAE) and mean absolute percentage error (MAPE) were calculated to quantify measurement error. On the basis of previous studies, a MAPE of ≤10% was considered acceptable [18,19]. The proportions of underestimation and overestimation were also calculated. All numerical values and statistical metrics were derived using CPX-measured HR as the reference standard. All analyses were conducted using R (version 4.4.0; R Foundation for Statistical Computing) and RStudio (version 2024.12.1+563; RStudio, Inc).

### Ethical Considerations

The study protocol complied with the principles of the Declaration of Helsinki (1975), as revised in 2000, and was approved by the institutional review boards of the Itami City Hospital (2537) and the Shijonawate Gakuen University (24‐4). Written informed consent was obtained from all participants after detailed explanations of the study’s objectives, procedures, potential benefits, and risks were provided by the researchers. To protect participant privacy, study records were de-identified. Each participant was assigned a unique study ID, and the linkage file connecting IDs to personal identifiers was stored exclusively on the hospital’s electronic medical record network; no identifiable information was transferred outside this network. Only variables necessary for the study were collected, and the datasets were processed and managed to prevent the immediate identification of any individual. All analyses were conducted using de-identified datasets on the local computers. Participants did not receive any financial or material compensation for participating in the study.

## Results

### Demographic and Clinical Characteristics

The demographic and clinical characteristics are summarized in Tables1  2. Of the 30 patients with CVD included in the study, 13 (43%) were classified as having stage B HF and 17 (57%) as having stage C HF. The median (IQR) age was 65 (54-73) years, and 17 patients (57%) were male. CAD was the most common underlying condition, observed in 16 patients (53%), followed by dilated cardiomyopathy in 6 (20%) patients. Regarding pharmacotherapy, β blockers were prescribed to 23 (77%) patients. According to the New York Heart Association classification, 8 (27%) patients were in class I; 20 (67%) in class II; and 2 (7%) in class III. The median (IQR) LVEF was 52% (45%‐61%), and 20% (6/30) of the patients were categorized as having HF with reduced ejection fraction. The median (IQR) B-type natriuretic peptide concentration was 56 (13‐93) pg/mL. During CPX, the total median exercise time was 8.33 (IQR 7.00‐10.45) minutes, the median peak respiratory exchange ratio was 1.16 (IQR 1.10‐1.23), and the median peak VO₂/BW was 17.2 (IQR 14.5‐21.2) mL/kg/min. When comparing the stage B (n=13, 43%) and stage C (n=17, 57%) groups, patients in the stage B group were older (median age 73 vs 55 y) and had a higher prevalence of CAD (11/13, 85% vs 5/17, 29%). In contrast, nonischemic etiologies, including dilated cardiomyopathy, were more common in the stage C group (6/17, 35% vs 0/13, 0%). β-blocker use was more frequent in the stage C group (17/17, 100% vs 6/13, 46%).

**Table 1.** Participant demographics and clinical profile.

Characteristics		Overall (n=30)	Stage B (n=13)	Stage C (n=17)
Age (y), median (IQR)		65 (54-73)	73 (64-78)	55 (48-65)
Male, n (%)		17 (57)	8 (62)	9 (53)
BMI (kg/m^2^), median (IQR)		23.2 (21.6-26.2)	22.0 (20.8-24.8)	23.7 (22.0-27.9)
Etiology, n (%)				
CAD^a^		16 (53)	11 (85)	5 (29)
DCM^b^		6 (20)	0 (0)	6 (35)
HHD^c^		2 (7)	0 (0)	2 (12)
Cardiac sarcoidosis		2 (7)	0 (0)	2 (12)
Others		4 (13)	2 (15)	2 (12%)
Comorbidities, n (%)				
Hypertension		19 (63)	10 (77)	9 (53)
Dyslipidemia		12 (40)	6 (46)	6 (35)
Smoking, n (%)				
Never		18 (60)	8 (62)	10 (59)
Past		8 (27)	4 (31)	4 (24)
Current		4 (13)	1 (8)	3 (18)
FH^d^, n (%)		1 (3)	1 (8)	0 (0)
DM^e^, n (%)		9 (30)	4 (31)	5 (29)
Respiratory disease, n (%)		4 (13)	2 (15)	2 (12)
LVEF^f^ (%), median (IQR)		52 (45-61)	54 (52-61)	46 (39-58)
LVEF classification, n (%)				
HFpEF^g^		16 (53)	10 (77)	6 (35)
HFmrEF^h^		8 (27)	3 (23)	5 (29)
HFrEF^i^		6 (20)	0 (0)	6 (35)
NYHA^j^ classification, n (%)				
1		8 (27)	4 (31)	4 (24)
2		20 (67)	9 (69)	11 (65)
3		2 (7)	0 (0)	2 (12)

a CAD: coronary artery disease.

b DCM: dilated cardiomyopathy.

c HHD: hypertensive heart disease.

d FH: family history.

e DM: diabetes mellitus.

f LVEF: left ventricular ejection fraction.

g HFpEF: heart failure with preserved ejection fraction.

h HFmrEF: heart failure with mildly reduced ejection fraction.

i HFrEF: heart failure with reduced ejection fraction.

j NYHA: New York Heart Association.

**Table 2.** Pharmacotherapy, laboratory biomarkers, and CPX^a^ parameters.

Characteristics		Overall (n=30)	Stage B (n=13)	Stage C (n=17)
ACE-i^b^, ARB^c^, or ARNI^d^, n (%)		28 (93)	13 (100)	15 (88)
β blocker, n (%)		23 (77)	6 (46)	17 (100)
SGLT2i^e^, n (%)		13 (43)	2 (15)	11 (65)
MRA^f^, n (%)		14 (47)	1 (8)	13 (76)
Diuretics, n (%)		7 (23)	0 (0)	7 (41)
Nitrates, n (%)		11 (37)	8 (62)	3 (18)
Calcium antagonists, n (%)		3 (10)	1 (8)	2 (12)
Antiplatelet agents, n (%)		16 (53)	12 (92)	4 (24)
Anticoagulants, n (%)		8 (27)	3 (23)	5 (29)
Statins, n (%)		16 (53)	11 (85)	5 (29)
Ezetimibe, n (%)		1 (3)	1 (8)	0 (0)
Amiodarone, n (%)		1 (3)	0 (0)	1 (6)
Hemoglobin (g/dL), median (IQR)		13.75 (11.83-14.98)	12.90 (11.80-14.80)	13.80 (12.30-15.00)
Creatinine (mg/dL), median (IQR)		0.92 (0.80-1.04)	0.88 (0.80-1.11)	0.92 (0.84-1.01)
eGFR^g^ (mL/min/1.73 m²), median (IQR)		62 (50-70)	61 (50-69)	64 (51-70)
BNP^h^ (pg/mL), median (IQR)		56 (13-93)	61 (25-114)	27 (12-83)
Missing BNP values, n (%)		1 (3)	1 (8)	0 (0)
Ramp protocol				
10 W, n (%)		26 (87)	12 (92)	14 (82)
20 W, n (%)		4 (13)	1 (8)	3 (18)
Total exercise time (min), median (IQR)		8.33 (7.00-10.45)	8.90 (7.00-10.60)	8.20 (7.15-9.65)
Peak RER^i^, median (IQR)		1.16 (1.10-1.23)	1.16 (1.11-1.23)	1.15 (1.04-1.22)
Peak WR^j^ (W), median (IQR)		93 (75-112)	93 (81-110)	98 (74-112)
Peak VO_2_^k^ (mL/min), median (IQR)		1158 (883-1324)	1097 (984-1,277)	1194 (820-1348)
Peak VO_2_/BW^l^ (mL/kg/min), median (IQR)		17.2 (14.5-21.2)	17.1 (15.6-22.1)	17.3 (14.4-20.2)
ATVO_2_^m^ (ml/min), median (IQR)		724 (610-823)	682 (624-772)	745 (584-840)
ATVO_2_/BW (mL/kg/min), median (IQR)		11.80 (9.86-12.82)	11.69 (10.64-12.54)	11.99 (9.52-12.83)

a CPX: cardiopulmonary exercise testing.

b ACE-i: angiotensin-converting enzyme inhibitor.

c ARB: angiotensin II receptor blocker.

d ARNI: angiotensin receptor–neprilysin inhibitor.

e SGLT2i: sodium-glucose cotransporter 2 inhibitor.

f MRA: mineralocorticoid receptor antagonist.

g eGFR: estimated glomerular filtration rate.

h BNP: B-type natriuretic peptide.

i RER: respiratory exchange ratio.

j WR: work rate.

k VO₂: oxygen uptake.

l VO₂/BW: oxygen uptake per body weight.

m ATVO₂: oxygen consumption at anaerobic threshold.

### Validity of HR Measurement Using the Fitbit Inspire 3

A comparison between HR measurements obtained via CPX and those estimated by the Fitbit Inspire 3 is presented. In total, 630 data points were analyzed and categorized into 4 exercise phases: rest, below and above the AT, and recovery (Table 3). These data were further stratified by HF stage (stages B and C; Table 4).

**Table 3.** Accuracy of Fitbit HR^a^ compared to CPX^b^-measured HR. A cutoff value of 10% for MAPE^c^ was adopted from previous studies to evaluate the error rate.

Condition	Correlation coefficient, *r* (95% CI)	MAE^d^ (bpm^e^), mean (SD)	MAPE (%), mean (SD)	Error rate, n (%)	Overestimation, n (%)	Underestimation, n (%)
All (N=630)^f^	0.90 (0.88‐0.91)	5.20 (8.75)	5.40 (8.33)	94 (15)	37 (6)	57 (9)
Rest (n=30)^f^	0.92 (0.84‐0.96)	3.40 (4.15)	4.67 (5.53)	3 (10)	2 (7)	1 (3)
Below AT^g^ (n=270)^f^	0.85 (0.82‐0.88)	4.41 (7.28)	5.23 (8.29)	39 (14)	10 (4)	29 (11)
Above AT (n=119)^f^	0.76 (0.67‐0.82)	8.03 (14.08)	6.14 (9.62)	20 (17)	1 (1)	19 (16)
Recovery (n=211)^f^	0.92 (0.90‐0.94)	4.85 (6.46)	5.32 (7.96)	27 (13)	22 (10)	5 (2)

a HR: heart rate.

b CPX: cardiopulmonary exercise testing.

c MAPE: mean absolute percentage error.

d MAE: mean absolute error.

e bpm: beats per minute.

f Number of data points.

g AT: anaerobic threshold.

**Table 4.** Accuracy of Fitbit HR^a^ compared to CPX^b^-measured HR stratified by heart failure stage. A cutoff value of 10% for MAPE^c^ was adopted from previous studies to evaluate the error rate.

Condition	Correlation coefficient, *r* (95% CI)	MAE^d^ (bpm^e^), mean (SD)	MAPE (%), mean (SD)	Error rate, n (%)	Overestimation, n (%)	Underestimation, n (%)
All (N=630)^f^	0.90 (0.88‐0.91)	5.20 (8.75)	5.40 (8.33)	94 (15)	37 (6)	57 (9)
Stage B (n=275)^f^	0.92 (0.90‐0.94)	4.51 (7.66)	4.67 (7.94)	31 (11)	17 (6)	14 (5)
Stage C (n=355)^f^	0.88 (0.85‐0.90)	5.73 (9.49)	5.98 (8.59)	58 (16)	18 (5)	40 (11)

a HR: heart rate.

b CPX: cardiopulmonary exercise testing.

c MAPE: mean absolute percentage error.

d MAE: mean absolute error.

e bpm: beats per minute.

f Number of data points.

**Figure 1.**
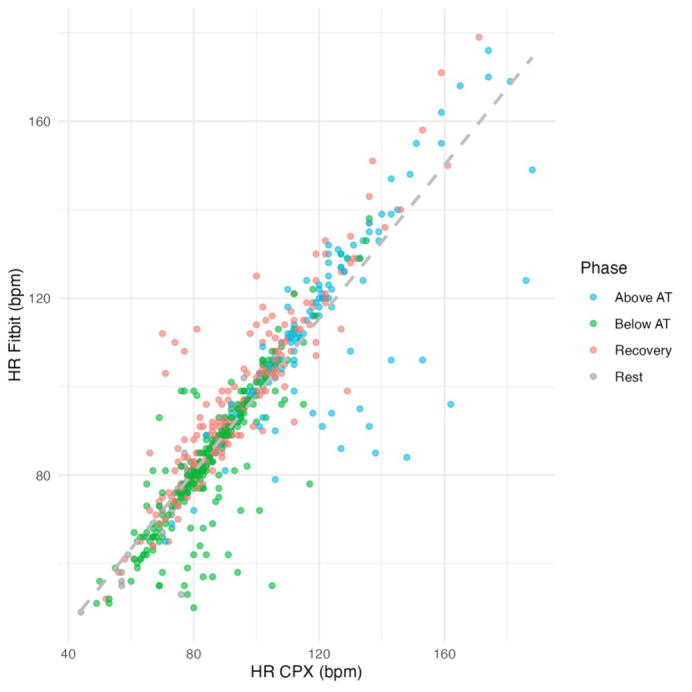
Scatter plot comparing heart rate (HR) measured by the Fitbit Inspire 3 with HR measured by cardiopulmonary exercise testing (CPX), plotted on the x-axis. Each point represents an individual measurement and is color-coded by exercise phase (rest, below anaerobic threshold [AT], above AT, and recovery). The dashed gray line indicates the line of identity (y=x). bpm: beats per minute.

**Figure 2.**
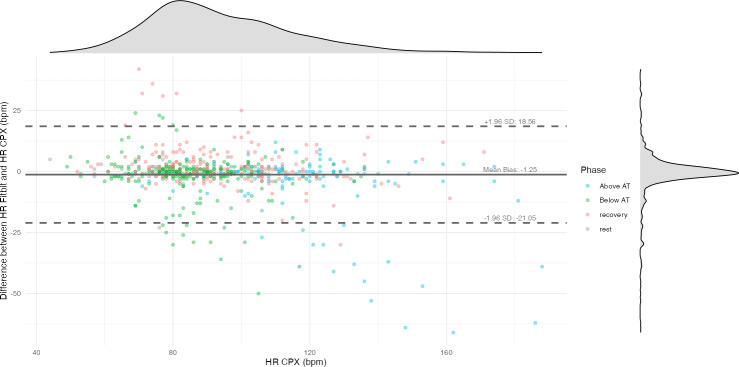
Bland-Altman plot comparing heart rate (HR) measurements from the Fitbit Inspire 3 and cardiopulmonary exercise testing (CPX). The x-axis represents HR measured by CPX, while the y-axis displays the difference between Fitbit and CPX HR values (Fitbit HR – CPX HR). The solid horizontal line indicates the mean bias (−1.25 beats per minute [bpm]), and the dashed lines represent the 95% limits of agreement (+18.56 bpm and −21.05 bpm). Density curves along the top and right margins show the distributions of CPX HR values and HR differences, respectively. Each point represents a single measurement and is color-coded by exercise phase (rest, below anaerobic threshold [AT], above AT, and recovery).

**Figure 3.**
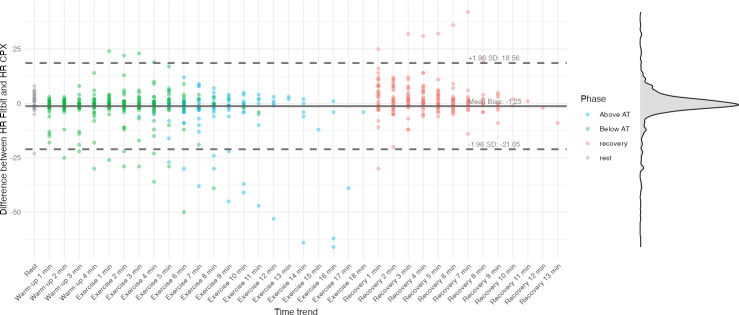
Time-series Bland-Altman plot comparing heart rate (HR) measurements from the Fitbit Inspire 3 and cardiopulmonary exercise testing (CPX). The x-axis represents time points across the CPX protocol, while the y-axis shows the difference in HR values (Fitbit HR – CPX HR). The solid horizontal line indicates the mean bias (−1.25 beats per minute [bpm]), and the dashed lines represent the upper (+18.56 bpm) and lower (−21.05 bpm) 95% limits of agreement. The density curve on the right illustrates the distribution of HR differences. Each point represents a single measurement, color-coded by exercise phase (rest, below anaerobic threshold [AT], above AT, and recovery).

In the overall sample (N=630), the correlation coefficient between CPX-measured HR and Fitbit Inspire 3–derived HR was 0.90 (95% CI 0.88‐0.91; Figure 1). The MAE was 5.20 (SD 8.75) beats per minute (bpm), and the MAPE was 5.40% (SD 8.33%). The total error was 94 (N=630, 15%), with overestimation and underestimation of 37 (6%) and 57 (9%), respectively. The Bland-Altman plot (Figure 2) displays CPX-measured HR on the x-axis and the difference between CPX and Fitbit HR measurements on the y-axis. The time-series trend of HR error, with time on the x-axis, is shown in Figure 3. The average difference in HR was −1.25 bpm, with upper and lower limits of agreement of 18.56 bpm and −21.05 bpm, respectively.

By condition, the rest phase (n=30) showed a correlation coefficient of 0.85 (0.82‐0.88), MAE of 4.41 (SD 7.28) bpm, and MAPE of 5.23% (SD 8.29%). During the below AT condition (n=270), the correlation coefficient was 0.92 (0.90‐0.94), MAE was 4.85 (SD 6.46) bpm, and MAPE was 5.32% (SD 7.96%).

In contrast, the above AT condition (n=119) demonstrated a lower correlation coefficient of 0.76 (0.67‐0.82), with higher MAE and MAPE values of 8.03 (SD 14.08) bpm and 6.14% (SD 9.62%), respectively. In the recovery phase (n=211), the correlation coefficient was 0.92 (0.90‐0.94), with an MAE of 4.85 (SD 6.46) bpm and a MAPE of 5.32% (SD 7.96%). Regarding estimation errors, the above AT phase exhibited a higher underestimation (19/119 points, 15.9%) than overestimation (1/119 points; 0.8%), whereas in the recovery phase, overestimation (22/211 points, 10.4%) exceeded underestimation (5/211 points, 2.3%).

By HF stage, stage B (n=275) demonstrated a correlation coefficient of 0.92 (0.90‐0.94) and an MAE of 4.51 (SD 7.66) bpm. In comparison, stage C (n=355) showed a slightly lower correlation coefficient of 0.88 (0.85‐0.90) and a higher MAE of 5.73 (SD 9.49) bpm. The over- and underestimations were 17 (N=275, 6.1%) and 14 (5%) for stage B and 18 (N=355, 5%) and 40 (11.2%) for stage C, respectively.

## Discussion

### Principal Findings

In this study, we analyzed the accuracy of HR measurements obtained from the Fitbit Inspire 3 relative to ECG-based HR during CPX in patients with CVD, including those with HF. The Fitbit Inspire 3 device yielded relatively accurate HR estimates at intensities below the AT. However, estimation errors increased above the AT: the device underestimated HR during high-intensity exercise and overestimated it during recovery. In addition, overall accuracy was lower in patients with HF.

### Comparison With Previous Work

The overall correlation coefficient was 0.90 (Table 2), whereas previous studies involving healthy individuals using Fitbit devices reported correlation coefficients ranging from 0.84 to 0.93 [20,21]. MAPE was 4.67% (SD 5.53%) at rest and 6.14% (SD 9.62%) above the AT. A study using the Fitbit Charge 4 across various activities, such as stair climbing and squats, found MAPE values ranging from 6.36% to 11.98%, depending on the activity [22]. Another cycling-based study using the Fitbit Charge 3 reported a MAPE of 6.1% [23]. Despite using the comparatively less-expensive Fitbit Inspire 3 in this study, we observed similar outcomes, suggesting that, if the exercise modality is equivalent, similar accuracy may be achieved.

As the workload increased during CPX, MAPE rose from 4.67% (SD 5.53%) at rest to 5.23% (SD 8.29%) below the AT and 6.14% (SD 9.62%) above the AT (Table 2). Correspondingly, measurement error increased with HR (Figure 2). A previous study that varied walking speeds on a treadmill reported MAPEs of 9.99% at 3.0 km/h and 10.06% at 6.4 km/h [20]. Another study that varied cycling workloads, similar to this study, found a MAPE of −7.0% at a light load (50 W) and −15% at workloads of 60%‐85% of HR reserve [24], indicating larger measurement errors at higher exercise intensities. The decreased accuracy at elevated workloads may be attributable to several factors: the low test-retest reliability of wrist-based PPG sensors (intraclass correlation coefficients <0.5) [25], increased forearm muscle contraction to stabilize the handlebars (which can reduce blood flow and introduce signal artifacts) [26,27], reduced contact between the PPG sensor and the skin [28], measurement latency [29], and motion artifacts themselves [30]. In our study, underestimation became more pronounced at higher exercise intensities, whereas overestimation predominated during the recovery phase (Figure 3). The acute phase of HR recovery is influenced by parasympathetic reactivation, and the late phase is associated with sympathetic withdrawal and reduced catecholamine levels, generally leading to a 12 to 30 bpm decrease within 1 minute [31]. The fact that 23 (77%) participants were taking β blockers suggests that pharmacological effects, autonomic dysfunction, and the Fitbit device’s delayed response to sudden changes in HR [24] may have collectively contributed to these discrepancies.

Notably, patients with stage C HF showed a higher rate of underestimation than those with stage B (11% vs 5%), likely owing to the larger measurement error at high workloads. No validation study has specifically focused on wearable devices in patients with HF. We speculate that because patients with HF have a limited ability to increase cardiac output beyond the AT [32] and experience reduced peripheral perfusion due to heightened sympathetic tone, PPG-based HR may be underestimated. Further research is warranted to clarify the accuracy of PPG-based HR measurements above the AT in this population.

### Limitations

This study has some limitations worth noting. First, it was a pilot study with a relatively small sample size. In addition, HR values were averaged over 1-minute intervals, leading to fewer data points. Consequently, rapid fluctuations in HR may not have been fully captured and could have contributed to measurement error. Second, we excluded patients with persistent AF; however, patients with both HF and AF may experience further reductions in HR measurement accuracy [33]. Third, we used cycling as the exercise modality, which restricted upper-limb movement. Home-based exercise therapies commonly involve walking, which requires arm movement. Therefore, we were unable to assess potential artifacts caused by arm motion. Further studies are needed to confirm the accuracy of these devices under more typical home-based exercise conditions. Fourth, the patients with HF in our cohort were relatively young and clinically stable. The results may differ in patients with more advanced HF, and caution should be exercised when generalizing these findings.

### Conclusions

This study demonstrated that the accuracy of HR estimation by the Fitbit Inspire 3 varied depending on exercise intensity and patient characteristics. These findings suggest that when using the Fitbit Inspire 3 to support interventions such as home-based exercise therapy in patients with CVD, including those with HF, careful consideration should be given to the patient’s HF stage and exercise intensity, as well as to the device’s potential limitations in different usage scenarios.

## Abbreviations

ACC: American College of Cardiology
AF: atrial fibrillation
AHA: American Heart Association
AT: anaerobic threshold
bpm: beats per minute
BW: body weight
CAD: coronary artery disease
CPX: cardiopulmonary exercise testing
CR: cardiac rehabilitation
CVD: cardiovascular disease
ECG: electrocardiography
HF: heart failure
HR: heart rate
LVEF: left ventricular ejection fraction
MAE: mean absolute error
MAPE: mean absolute percentage error
PPG: photoplethysmography
VCO_2_: carbon dioxide output
VE: minute ventilation
VO_2_: oxygen uptake
